# Presence of Chlorate and Perchlorate Residues in Raw Bovine Milk from Italian Farms

**DOI:** 10.3390/foods11182741

**Published:** 2022-09-06

**Authors:** Maria Nobile, Luigi Danesi, Radmila Pavlovic, Giacomo Mosconi, Federica Di Cesare, Francesco Arioli, Roberto Villa, Luca Maria Chiesa, Sara Panseri

**Affiliations:** Department of Veterinary Medicine and Animal Science, University of Milan, Via dell’Università 6, 26900 Lodi, Italy

**Keywords:** milk, chlorate, perchlorate, IC-HRMS, risk characterization, food safety

## Abstract

Chlorates and perchlorates are endocrine disruptors and emerging environmental contaminants found in various types of foods, including milk and dairy products. The presence of chlorate has been associated with the use of chlorine-based sanitizers to disinfect equipment and water used in food processing. Perchlorate, on the other hand, occurs naturally in the environment but is also released from anthropogenic sources. To protect consumers, the EU set an MRL for chlorate in milk but not for perchlorate. Considering that data on chlorates and perchlorates in this field are limited, the objective of this study was to assess the presence of these two anions in 148 samples of raw bovine milk collected from different farms of Lombardy and grouped in three different geographical zones. Chlorate was detected in 73% of the samples analyzed, at concentrations ranging from <LOQ to 18.70 μg kg^−1^ with an average value of 7.10 ± 5.88 μg kg^−1^ below the MRL; perchlorate with a frequency of 99%, in the range from <LOQ to 6.95 μg kg^−1^ and an average value of 4.06 ± 1.58 μg kg^−1^. No significant differences were detected among the three geographical zones. An evaluation of the estimated daily intake of perchlorate through milk confirmed the absence of risk for Italian consumers.

## 1. Introduction

The quality of milk and dairy products begins on the farm, and especially, in the milking room. Milking room behavior, techniques, cleaning and disinfection methods, as well as milking routines, can change the quality characteristics of milk [1]. The increase in bacteria in milk causes a deterioration in quality, because the transformations they carry out, accumulate and transfer loss of taste to the milk and produce enzymes that destroy the primary components, resulting in lower yields and poorer quality cheeses [2].

The cleaning and disinfection of equipment used for the transfer and storage of milk and the manufacture of dairy products is important to ensure a high standard of food hygiene. Chlorine-based disinfectants are commonly used during the dairy processing chain because of their efficiency in the washing and sanitation of contact surfaces and their relatively low price [3].

Chlorate is not permitted as a pesticide in the European Union (EU) according to Commission Decision No 2008/865/EC [4]. It is well known that chlorate formation occurs as a by-product after the use of hypochlorite, chlorine or chlorine dioxide to disinfect drinking water, water destined for food production and surfaces in contact with food. Chlorination of food of animal origin is not permitted in the EU, while the washing of plants for food production with chlorine disinfected water can be allowed under national regulations.

Chlorate in food was revealed in 2014 during an official control by chance and in 2015, the European Food Safety Authority (EFSA, Parma, Italy) notified that chlorate concentrations in both drinking water and foods were too high, resulting in potentially serious health implications as well as the alteration of thyroid function with the inhibition of iodine uptake, especially among infants and children. With the Regulation (EC) 396/2005 [5], the default maximum residue level (MRL) of 0.01 mg kg^−1^ was recommended for all food products. Considering that the chlorate concentration in different kinds of food exceeded this default, new enforcement measures on the default level were considered by the EU. Through a multi-disciplinary action plan started in 2017, different points were defined: setting a chlorate MRL in drinking water, suggesting good hygiene practices to reduce chlorate formation from chlorinated disinfectants; keeping the MRLs at 0.01 mg kg^−1^ for food intended for infant and children and establishing MRLs in other food categories on the basis of data collected by member states, as finalized in the new Commission Regulation (EU) 2020/749 [6] applied since June 2020. On this purpose, an MRL of 0.1 mg kg^−1^ was established for milk, including raw milk, heat-treated milk and milk intended for the manufacture of milk-based products as defined in the Regulation (EU) No. 1308/2013. The MRL applies to milk ready for consumption (and marketed as such or reconstituted according to the manufacturer’s instructions).

Regarding perchlorates, they are contaminants of natural and anthropogenic origin present in the environment. Due to their strong oxidizing power, the uses of perchlorates in the industry are different: they can be used in the military field, but also in the production of food contact materials (MOCA) as additives or auxiliary substances of polymerization. They are stable and water-soluble so they can be present in water, soil, and then absorbed by plants and contaminate food, especially fruit and vegetables. Perchlorate contamination of food of plant origin is often associated with the use of inorganic fertilizers, resulting from their natural presence in the raw materials used for their production. Moreover, perchlorate is one of the degradation products originating from chlorinated disinfectants used for water processing. It could be also formed by photochemical reactions in the atmosphere and released from some plant protection products containing chlorine. Concerning perchlorate, the EFSA Panel on Contaminants in the Food Chain (CONTAM Panel) confirmed the conclusion that the current chronic, short-term exposure to perchlorate may be a possible human health concern, especially for some more vulnerable groups, such as infants. In the literature, there are few works (Table 1) in this regard, considering one of the analytes or mostly on infant formula and breast milk, with analytical protocols different from those proposed in this study.

Considering the years affected by the COVID-19 pandemic, during which disinfection habits have increased in every context of daily life, the aim of the present work was to study the perchlorate and chlorate contaminations in raw bovine milk, a raw material then used for myriad uses in the dairy industry, which best represents the contamination situation related to disinfection practices at the farm.

## 2. Materials and Methods

### 2.1. Chemicals and Reagents

All solvents and reagents were from Merck (Darmstadt, Germany). Water was purified by a Milli-Q system (Millipore, Merck, Darmstadt, Germany). Chlorate, perchlorate and the stable-isotope labeled version of chlorate (Cl^18^O_3_^−^), as internal standard, were obtained by LGC Standards (Milan, Italy).

### 2.2. Standard Solutions

The stock solutions (1 mg mL^−1^) were prepared in water and stored at −20 °C. The working solutions at 10 and 100 ng mL^−1^ were prepared in water and kept at 4 °C.

### 2.3. Sample Collection

One hundred and forty-eight raw bovine milk samples were randomly collected from farms of the 12 Lombardy provinces (Italy) grouped into 3 geographical zones: 61 samples were from Po Valley, 28 from the Alpine zone and 59 from the Sub-alpine one. Each sample was collected into bottles of 500 mL, then stored at −20 °C until the analysis.

### 2.4. Sample Extraction

Sample extraction was performed according to the protocol described in our previous work [13]. Briefly, 1 mL of milk was spiked with the internal standard (IS) at the final concentration of 10 ng mL^−1^ and extracted with 10 mL of a mixture of 30:70 methanol/1% formic acidified water. After the vortex, sonication for 15 min and centrifugation (4 °C, 10 min, 2500× *g*), 1 mL of the supernatant was filtered in a vial.

### 2.5. IC-HRMS Orbitrap Parameters

All the details and parameters about ion cromatography–high resolution mass spectrometry (IC-HRMS) analysis are described in Chiesa et al. [14], slightly modified for the part of high-resolution mass spectrometry for the use of new Exploris™ 120 detector (Thermo Scientific, San Jose, CA, USA) for this application. Briefly, the IC compartment consisted of a Dionex ICS-5000 + system (Sunnyvale, CA, USA) made up of an Autosampler, a Dual Pump and a Conductivity Detector. The column, kept at 30 °C, was a Thermo Scientific Dionex IonPac AS19-4 μm (2 × 250 mm, 4 μm particle size) with a guard column Dionex IonPac AG19-4 μm (2 × 50 mm). The suppressor was a Dionex AERS 144 500, 2 mm (Thermo Scientific, San Jose, CA, USA). Chromatographical separation started at 15 mM KOH (aq) kept for 8 min, and then increased linearly up to 55 mM KOH (aq) at the 20th min, and was kept in this condition for 4 min. After 1 min, the initial KOH concentration was restored and kept until the 30th min. The water flow rate was set at 0.25 mL/min and the injection volume was 50 µL.

**Figure 1:**
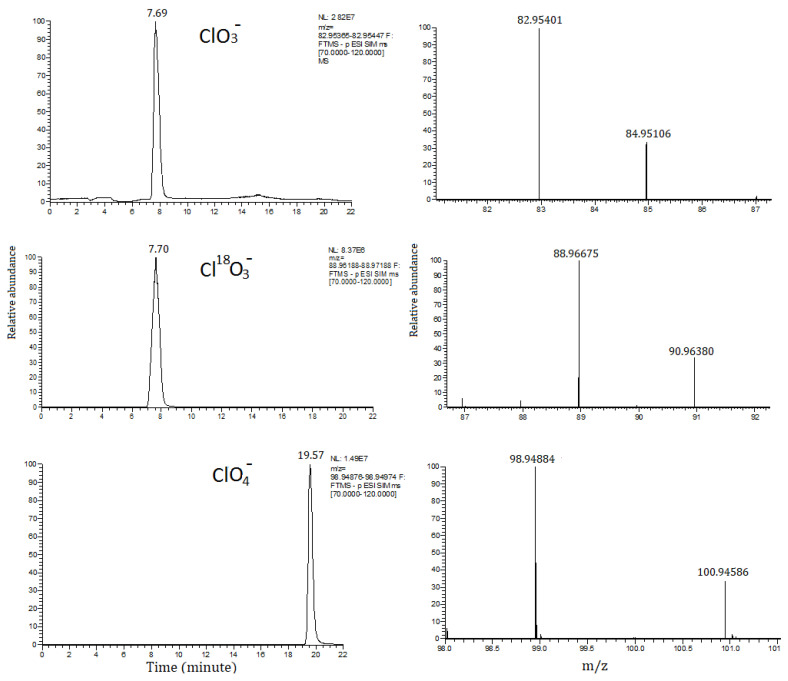
Extracted ion chromatograms and related mass spectra of chlorate, chlorate ^18^O (as internal standard), and perchlorate anions in a blank sample spiked at 10 ng g^−1^ along with a characteristic isotopic pattern used for conformation purpose.

An Orbitrap Exploris™ 120 high-resolution mass spectrometer (Thermo Scientific, San Jose, CA, USA), equipped of a heated electrospray ionization source (HESI, Washington, DC, USA) operating in negative mode was used. Instrument calibration was carried out every analytical session with a direct infusion of a Pierce FlexMix Calibration solution (Thermo Scientific, San Jose, CA, USA). Capillary temperature and vaporizer temperature were set at 330 °C and 280 °C, the HESI spray voltage was 3 kV; sheath and auxiliary gas were set at 35 and 15 arbitrary units, respectively. The full scan acquisition mode was selected, with a resolution of 60,000 FWHM, a scan range of *m*/*z* 70–120, an automatic gain control (AGC) set in the standard mode, with an automatic maximum injection time and an RF lens level of 70% and microscans of 1. For the confirmation of ClO^3−^ and ClO^4−^, in addition to the parent ion identification, the isotopic pattern of naturally occurring ^37^Cl was also checked in the full scan acquisition, as previously reported [13], and shown in Figure 1, for a blank sample spiked at 10 ng g^−1^.

### 2.6. Method Validation Assessment

Validation was carried out according to the Guidance SANTE 11312/2021 [15] on analytical quality control and method validation procedures for pesticide residues analysis in food and feed. Moreover, the method follows the US Food and Drug Administration Foods Veterinary Medicine Research Steering Committee (US FDA FVM) recommendations for the confirmation of the identity of compound residues using exact mass data [16].

The method was validated for selectivity/specificity, recovery, precision, linearity and sensitivity with a limit of quantification (LOQ) determination. The requirements for the identification of analytes were: a S/N ≥ 3, the relative intensities or ratios of selective ions, expressed as a ratio relative to the most intense ion, used for identification, which matched with the reference ion ratio. A number of six replicates were analyzed to check the recovery and precision at two concentration levels (LOQ and 10 ng g^−1^). The LOQ was the lowest validated spiked level meeting the identification and method performance criteria for recovery (70–120%) and precision (RSD ≤ 20%). The matrix effect, expressed as percentage, was also assessed by comparing the response of analytes in the solvent solution with that of analyte in the sample extract, in the same amount. The matrix-matched calibration curves were made of 5 calibration points in triplicate at 0.5, 10, 20, 50 and 100 ng g^−1^ for the 2 analytes, covering the concentration range found in the samples.

### 2.7. Statistical Analysis

Statistical analyses were carried out using Sigma Stat (StatisticalAnalysis System, version 12.5) software (Jandel Scientific GmbH, Erkrath, Germany). The Kolmogorov and Smirnov test was first performed on the entire number of samples to test whether the distribution of the concentration values of the two anions was normal or not, with *p* > 0.05. Since not all distributions were normally distributed, a non-parametric ANOVA (Kruskal–Wallis test) was carried out to compare the distributions.

## 3. Results and Discussion

### 3.1. Validation Performances

All validation parameters are reported in Table 2. Briefly, the method showed high specificity, with no interference close to the retention time of chlorate and perchlorate ions. The obtained LOQ values showed high sensitivity; the satisfactory recoveries were within the acceptable ranges set by the guidelines, the CVs % less than 20% and the matrix effect showed a slight enhancement of the signal but in general less than 20% as suggested by SANTE 11312/2021 [15]. Finally, a good linearity was obtained from LOQ to 100 ng g^−1^ with R^2^ higher than 0.99.

### 3.2. Analysis of the Samples

**Figure 2:**
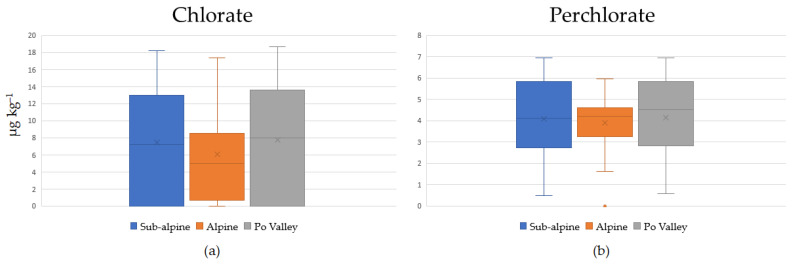
Average concentrations (μg kg^−1^) of chlorate (**a**) and perchlorate (**b**) in samples according to the 3 different geographical zones (Po Valley, Alpine and Subalpine).

Perchlorate was detected in 99% of the samples analyzed in the range from <LOQ to 6.95 μg kg^−1^ with an average concentration value of 4.06 ± 1.58 μg kg^−1^. Among the 12 provinces of Lombardy, grouped into three geographical zones (Po Valley, Alpine and Subalpine), as is shown in Table 3, no significant difference in perchlorate ion content was detected in the distributions (Figure 2). Perchlorate detected in such a high frequency implies that dairy cows have taken up this anion through feed and water. In particular, this presence derives both from natural formation in the atmosphere and surface water, and from anthropogenic sources including from inorganic fertilizers and industrial emissions, such as the use of ammonium perchlorate in solid rocket and missile propellants and from the degradation of chlorinated disinfectants used for water processing [8]. The values found in the milk samples used for this study are in line with the data brought in the Contaminant Panel report [17] for this food category. The most likely hypothesis explaining the presence of perchlorate in milk is that perchlorate is actively transported through the sodium/iodide symporter (NIS) during milk formation at the mammary level. The concentration of perchlorate in milk is directly proportional to the concentration of perchlorate present in the food [8]. In order to accelerate the clearance of perchlorate, metabolically, it is possible to increase the number of daily milkings, which must be more than two to see a significant change in concentrations. Iodine-based immersion disinfectants can also be used at the milking stage to decrease perchlorate reduction reactions, thereby decreasing the concentration of perchlorate in marketed milk [18]. It is also expected that, by reducing the intake of perchlorate through contaminated feed and water for feeding cow, the risk of perchlorate intake for humans through milk consumption may be reduced [8].

Chlorate was detected in 73% of the samples analyzed in the range from <LOQ to 18.70 μg kg^−1^ with an average concentration value of 7.09 ± 5.87 μg kg^−1^. Additionally, in this case, among the 12 provinces of Lombardy, grouped into three geographical zones (Po Valley, Alpine and Subalpine), no significant difference in its distributions was detected (Figure 2). Considering the legal limits (100 μg kg^−1^) according to EU Regulation 2020/749, all the analyzed samples showed full compliance.

Chlorate enters the supply chain as a consequence of its presence in drinking water but mostly as a disinfection byproduct, either through the contact between milk with chlorinated water or as a residue from cleaning-in-place processes present on equipment surfaces.

This also shows that farms have adopted and followed all recommendations regarding the disinfection of equipment used for raw milk production.

As reported in the introduction, it was seen that the potential for contamination starts as early as the farm level, where disinfection procedures, especially during the milking phase, are strongly influenced by the farmer and their adherence to specific hygiene protocols and good production practices [19].

Sanitation practices that are properly carried out on the farm help to keep chlorate ion concentration low [10]. Therefore, precautionary measures in disinfection during milking must be implemented, especially in this historical period characterized and affected by the global pandemic of COVID-19.

### 3.3. Risk Characterization on Perchlorate

In 2014, the Panel of Scientific Experts on Contaminants (CONTAM) of the European Food Safety Authority issued a Scientific Opinion on the public health risks associated with the presence of perchlorate in food, recommending a tolerable daily intake (TDI) of 0.3 μg kg^−1^ body weight per day, based on the inhibition of thyroid iodine uptake in healthy adults, specifying that the continued consumption of foods with high concentrations of perchlorate poses a public health risk, especially in younger consumers with iodine deficient [20].

The per capita consumption of fresh milk, which was historically approximately 50 L per year, has declined by 25–30% in the last five years. Today, an Italian consumer drinks approximately 115 mL a day. Thus, considering the highest average concentration detected (4.16 μg kg^−1^) in the Po Valley raw bovine milk samples, we calculated the estimated daily intake (EDI) as follows:

EDI = C × DC/BW,(1)

where C is the perchlorate average concentration mentioned above, DC is the daily milk consumption for the Italian population considering the density of raw cow’s milk (1.030 kg L^−1^) and BW is the consumer body weight (70 kg). The calculated EDI is 0.007 μg kg^−1^ body weight per day, a value constituting the 2% of the TDI, which leads to milk being of no concern with regard to perchlorate content. 

## 4. Conclusions

Taking in consideration the results of the study, it is evident that chlorates and perchlorates were found in a high occurrence in raw bovine milk samples. Briefly, chlorates were found in 73% of samples at concentrations ranging from <LOQ to 18.70 μg kg^−1^ with an average value of 7.10 ± 5.88 μg kg^−1^, below the MRL set by Regulation (EU) No. 2020/749. Perchlorate was almost ubiquitous, with 99% of occurrence in the samples analyzed with concentrations ranging from <LOQ to 6.95 μg kg^−1^ and an average value of 4.06 ± 1.58 μg kg^−1^.

By dividing the samples from the 12 Lombardy provinces into 3 geographical zones (Po Valley, Subalpine and Alpine), no significant differences in the detected concentrations of chlorates and perchlorates were found. Probably, the absence of a statistically significant difference was due to the fact that the samples were all collected from medium-sized and large dairy cattle farms, also denoting a proper application of good hygienic practices in animal husbandry, especially considering the particular historical period of the pandemic situation from COVID-19, which strongly implemented disinfection habits in daily life.

The study started with dairy cow farms because these represent the starting point for contamination in dairy food products from the raw material. Chlorates are mainly derived from the sanitization and disinfection processes that are applied on the farm: in recent years, thanks to the application of the good manufacturing practices of the multidisciplinary action plan of the member states, the values of chlorates presented in the literature have significantly improved, with a general trend toward decreasing levels. This is reflected in the analyzed samples, which have shown no health risk to the consumer.

## Figures and Tables

**Table 1:** State of the art of extraction techniques, instrumental analysis, limits of the methods and applications the on detection of chlorate and perchlorate in milk.

Reference	Analyte	Matrix	Extraction Technique	Instrumental Analysis	Limits of the Method (ng g^−1^)	Application Range Concentration (ng g^−1^)
[7]	Perchlorate	47 dairy milk, 36 breast milk and other matrices from China	Extracton with deionized water and acetonitrile, centrifugation, filtration	IC-MS/MS	MDL 10	0.30–9.1
[8]	Perchlorate	37 dairy milk and 26 milk-based powdered infant formula samples from South Korea	Extraction with ACN + 1% acetic acid + ethanol, centrifugation, SPE C-18, filtration	IC-MS/MS	LOQ 0.12–1.0	1.49–33.3
[9]	Perchlorate	milk powder and milk from China	Extraction with 1% acetic acid and acetonitrile, centrifugation, Strata X SPE, filtration	HILIC-MS/MS	LOD 4–8	1.32–4.10
[10]	Perchlorate and chlorate	67 mid- and late-lactation milk samples from Ireland	QuPPe method	LC/MS-MS	LOQ 1–10	Chlorate 1–155
[11]	14 highly polar pesticides	plant-based milk, wine and beer	Automatic extraction with mixture of acetonitrile/water, 6:4, *v*/*v*, containing 0.2% trifluoroacetic acid, centrifugation, dilution	LC/MS-MS	LOQ 10	No application
[12]	Perchlorate and chlorate	62 breast milks, 53 infant formulas, 88 baby supplementary food and 50 tap water samples from South China	Ultrasonic extraction with a mixture of 5.0 mL 0.1% formic acid and 10.0 mL methanol, centrifugation, PRiME HLB SPE cartridge, filtration	LC-MS/MS	LOD 0.03 and 0.12 LOQ 0.10 and 0.40 for perchlorate and chlorate, respectively	Perchlorate 0.56–1.18 Chlorate 1.73–2.67

IC-MS/MS: Ion chromatography–tandem mass spectrometry; HILIC-MS/MS: Hydrophilic interaction liquid chromatography–tandem mass spectrometry; LC/MS-MS: Liquid chromatography–tandem mass spectrometry; MDL: Method detection limit; LOD: Limit of detection; LOQ: Limit of quantification.

**Table 2:** Exact mass and validation performances (LOQ, matrix effect, precision, repeatability, recovery and linearity) of the method for chlorate and perchlorate in raw bovine milk.

	Exact Mass *m*/*z*	LOQ μg kg^−1^	Matrix Effect %	CV Intra-Day %	CV % Inter-Day %	Recovery %	Linearity R^2^
Chlorate	82.95406	0.5	112	11	15	79	0.9921
Perchlorate	98.94925	1	108	7	12	92	0.9981

LOQ: limit of quantification; CV: coefficient of variation.

**Table 3:** Occurrence of perchlorate and chlorate in raw bovine milk according to the 3 geographic areas of Lombardy. The total samples, number of positives, average, minimum and maximum concentrations (μg kg^−1^) are reported.

Perchlorate	Sub-Alpine Zone	Alpine Zone	Po Valley Zone
Total samples	59	28	61
N° of Positives (%)	59 (100%)	27 (96%)	61 (100%)
Average (μg kg^−1^) ± SD	4.10 ± 1.68	3.92 ± 1.39	4.16 ± 1.68
Min–Max (μg kg^−1^)	0.50–6.95	<LOQ–5.97	0.58–6.94
Chlorate			
Total samples	59	28	61
N° of Positives (%)	43 (73%)	21 (75%)	43 (70%)
Average (μg kg^−1^) ± SD	7.44 ± 6.00	6.09 ± 5.24	7.76 ± 6.39
Min–Max (μg kg^−1^)	<LOQ–18.22	<LOQ–17.40	<LOQ–18.70

## Data Availability

All related data and methods are presented in this paper. Additional inquiries should be addressed to the corresponding author.
